# Mechanism of Topotactic Reduction-Oxidation Between Mg-Doped SrMoO_3_ Perovskites and SrMoO_4_ Scheelites, Utilized as Anode Materials for Solid Oxide Fuel Cells

**DOI:** 10.3390/ma18153424

**Published:** 2025-07-22

**Authors:** Vanessa Cascos, M. T. Fernández-Díaz, José Antonio Alonso

**Affiliations:** 1 Department of Energy, CIEMAT, Av. Complutense 40, 28040 Madrid, Spain; 2 Institut Laue Langevin, BP 156X, F-38042 Grenoble, France; ferndiaz@ill.fr; 3 Instituto de Ciencia de Materiales de Madrid, Consejo Superior de Investigaciones Científicas (C.S.I.C.), Cantoblanco, 28049 Madrid, Spain

**Keywords:** scheelite structure, SrMoO_4_, oxygen vacancy, reversibility, SrMoO_3_, SOFC, solid oxide fuel cells, anode material

## Abstract

Recently, we have described SrMo_1-x_Mg_x_O_3-δ_ perovskites (x = 0.1, 0.2) as excellent anode materials for solid oxide fuel cells (SOFCs), with mixed ionic and electronic conduction (MIEC) properties. After depositing on the solid electrolyte, they were annealed for sintering at high temperatures (typically 1000 °C), giving rise to oxidized scheelite-type phases, with SrMo_1-x_Mg_x_O_4-δ_ (x = 0.1, 0.2) stoichiometry. To obtain the active perovskite phases, they were reduced again in the working anode conditions, under H_2_ atmosphere. Therefore, there must be an excellent reversibility between the oxidized Sr(Mo, Mg)O_4-δ_ scheelite and the reduced Sr(Mo, Mg)O_3-δ_ perovskite phases. This work describes the topotactical oxidation, by annealing at 400 °C in air, of the SrMo_0.9_Mg_0.1_O_3-δ_ perovskite oxide. The characterization by X-ray diffraction (XRD) and neutron powder diffraction (NPD) was carried out in order to determine the crystal structure features. The scheelite oxides are tetragonal, space group *I4_1_/a* (No. 88), whereas the perovskites are cubic, s.g. *Pm-3m* (No. 221). The Rietveld refinement of the scheelite phase from NPD data after annealing the perovskite at 400 °C and cooling it down slowly to RT evidences the absence of intermediate phases between perovskite and scheelite oxides, as well as the presence of oxygen vacancies in both oxidized and reduced phases, essential for their performance as MIEC oxides. The topotactical relationship between both crystal structures is discussed.

## 1. Introduction

Solid oxide fuel cells (SOFCs) are electrochemical devices that produce clean and efficient energy to feed electrical power devices [1]. They can use different types of fuels and possess the benefits of environmentally benign power generation [2,3]. Perovskite oxides with formula ABO_3_ are a consolidated class of electrode materials suitable for energy conversion devices due to their different structures, chemical composition, and high chemical stability [4]. They are typically synthesized via solid-state reactions or soft chemistry procedures and they may perform as both electrodes, working in the presence of air to catalyze the oxygen reduction reaction (ORR) as cathode materials or in fuel oxidation reaction as anodes. In this case, the stability in a reducing atmosphere is a requisite, since they typically work in the presence of H_2_ as a fuel at high temperatures [5,6,7].

A successful type of perovskite oxide stable in H_2_ atmosphere is the recently reported SrMo_1-x_M_x_O_3-δ_ (M = Fe, Ru, Mg and Ga; x = 0.1 and 0.2) perovskite phases [8,9,10,11], which outperform as anode materials for solid oxide fuel cells (SOFCs). These oxides are derivatives of SrMoO_3_ perovskite (containing Mo^4+^ ions), and they can be synthesized via the conversion of scheelite-type SrMoO_4_ (containing Mo^6+^) oxides upon thermal treatment under a reducing atmosphere. These perovskites are mixed ionic and electronic (MIEC) conductors resulting from a combination of the metallic conductivity of the pristine SrMoO_3_ oxide, together with the effect of doping with trivalent or divalent elements (M = Fe, Cr, Ga, Mg) at the Mo^4+^ positions, which promotes the presence of oxygen vacancies at the working temperatures of SOFCs (700–850 °C) [12,13,14,15].

The reversibility of the scheelite (oxidized) and perovskite (reduced) oxides is an essential requisite for the cycling and performance of the fuel cells; for that reason, in this work we describe the topotactical oxidation of oxygen-deficient SrMo_1-x_M_x_O_3-δ_ (M = Mg; x = 0.1) perovskite oxide to the corresponding scheelite phase. The aim of this work is to investigate by neutron powder diffraction (NPD) the perovskite-scheelite transformation, by heating in air the perovskite at 400 °C (temperature where the transformation of the perovskite to scheelite oxide starts to take place) and then, cooling down the sample to RT where the NPD measurement were carried out, thus quenching a mixture of the oxidized and reduced oxides where to investigate the structural features during the topotactical transformation and the possible presence of intermediate phases.

## 2. Experimental

SrMo_0.9_Mg_0.1_O_4-δ_ oxide was prepared as polycrystalline powders from citrate precursors obtained by soft-chemistry procedures. Stoichiometric amounts of Sr(NO_3_)_2_ (99%; Sigma-Aldrich, St. Louis, MI, USA), (NH_4_)_6_Mo_7_O_24_·4H_2_O (99.99%; Sigma-Aldrich) and Mg(NO_3_)_2_·6H_2_O (99%; Sigma-Aldrich), were dissolved in citric acid. By gentle heating, the water evaporation leads to an organic resin with the metal ions distributed at random. The resin was decomposed at 600 °C for 12 h in air, which gave rise to a pure scheelite oxide, with a moderate crystallinity. This scheelite precursor powder was then reduced in forming gas (5%H_2_/95%N_2_) flow at 1050 °C for 15 h, to obtain a pure SrMo_0.9_Mg_0.1_O_3-δ_ perovskite phase. Finally, the perovskite phase was re-oxidized at 400 °C in air for 1 h to obtain a mixture of the SrMo_0.9_Mg_0.1_O_4-δ_ scheelite oxide and the SrMo_0.9_Mg_0.1_O_3-δ_ perovskite oxide when the topotactical transformation is happening.

The initial characterization of the product was carried out by XRD with a Bruker D8 Advanced diffractometer (40 kV, 30 mA) (Bruker Corporation, Madrid, Spain), controlled by a DIFFRACT^PLUS^ software, version 6.x, in Bragg-Brentano reflection geometry with Cu K_α_ radiation (λ = 1.5418 Å) and a PSD (Position Sensitive Detector). For the structural refinement, NPD data were collected in the diffractometer D2B at ILL (Grenoble, France). The high intensity mode (Δd/d ≈ 5·10^−4^) was selected, with a neutron wavelength λ = 1.594 Å within the angular 2θ range from 8° to 150°. About 2 g of the sample were contained in a vanadium can. The measurement was carried out in air at 25 °C. The collection time was of 3 h per pattern. The NPD data were analyzed by the Rietveld method [16] with the FULLPROF program, version December 2008 [17]. A pseudo-Voigt function was chosen to generate the line shape of the diffraction peaks. The following parameters were refined in the final run: scale factor, background coefficients, zero-point error, pseudo-Voigt corrected for asymmetry parameters, positional coordinates and isotropic thermal factors for all the atoms. The coherent scattering lengths for Sr, Mg, Mo, and O were 7.02, 5.375, 6.715, and 5.803 fm, respectively.

## 3. Results and Discussion

### Crystallographic Characterization

**Figure 1:**
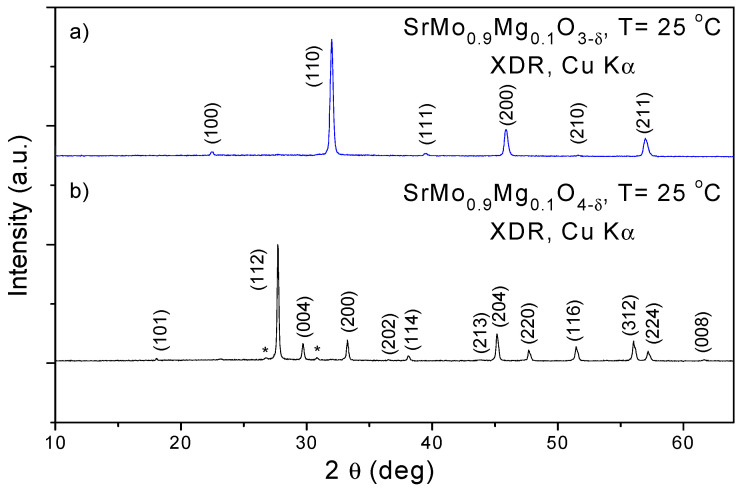
XRD patterns of (**a**) Sr(Mo, Mg)O_3_ perovskite and (**b**) Sr(Mo, Mg)O_4_ scheelite precursor. The asterisk in Figure 1b corresponds to a tiny impurity of Sr_3_MoO_6_.

The SrMo_0.9_Mg_0.1_O_4-δ_ oxide obtained by direct treatment in air of the citrate precursors was identified as a scheelite phase, as illustrated in the indexed XRD diagrams shown in Figure 1b. The asterisk in Figure 1b corresponds to a tiny impurity of Sr_3_MoO_6_ very typical in these types of materials [9]_._ After the thermal treatment at 1050 °C in 5% H_2_, the scheelite oxide is reduced to a perovskite oxide, with the characteristic XRD patterns displayed in Figure 1a, which correspond to a cubic phase with a = a_0_ = 3.9739 (4) Å at RT. Perovskite SrMo_0.9_Mg_0.1_O_3-δ_ patterns correspond to pure and well-crystallized single-phase oxide meaning that the tiny impurity of Sr_3_MoO_6_ disappeared after the second thermal treatment in 5% H_2_.

It is worth commenting that our strategy to obtain a MIEC oxide starting from metallic SrMoO_3_ perovskite is the chemical doping with aliovalent Mg^2+^ ions at the Mo-site, thus creating oxygen vacancies given the lower valence of Mg with respect to Mo. Mg^2+^ ions are introduced into the octahedral positions of the perovskite structure and, as an additional benefit, an expansion of the unit-cell dimensions is produced due to its large ionic size (0.72 Å) [18]. These features promote the ionic diffusion of oxide ions across the solid. Moreover, Mg^2+^ ions and LSGM electrolyte are completely compatible, since LSGM contains this element. In this work, 10% Mg^2+^ was successfully introduced into the perovskite structure, creating a new MIEC material that can be used as fuel electrode in SOFCs working at intermediate temperatures, in the 750–850 °C range. The excellent performance of SrMo_0.9_Mg_0.1_O_3-δ_ oxides as anodes has been reported elsewhere [10].

In this work, we were interested in the structural evolution of the sample, to disclose the perovskite-scheelite transformation by re-oxidizing the perovskite (reduced phase) sample in air at 400 °C and cooling it down to RT. A NPD study was essential to unveil some structural features related to the oxygen positions and occupancy.

**Figure 2:**
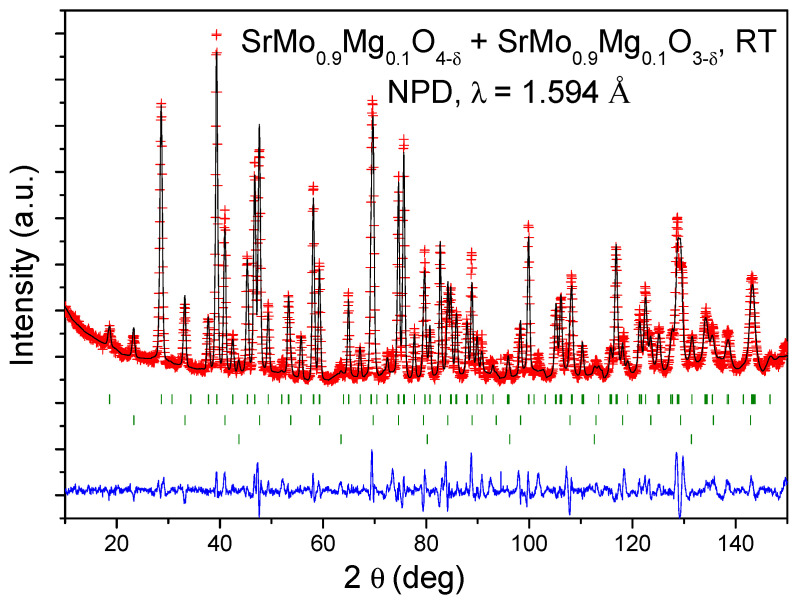
Observed (crosses), calculated (full line), and difference (blue line at the bottom) NPD profiles of the mixture of SrMo_0.9_Mg_0.1_O_4-δ_ scheelite and SrMo_0.9_Mg_0.1_O_3-δ_ perovskite, coexisting after heating the SrMo_0.9_Mg_0.1_O_3-δ_ perovskite sample at 400 °C for 1 h and cooling down to RT. The three series of reflection markers correspond to the SrMo_0.9_Mg_0.1_O_4-δ_ scheelite (first series), SrMo_0.9_Mg_0.1_O_3-δ_ perovskite (second series) and V from the sample holder (third series).

After heating at 400 °C in air conditions for 1 h and cooling down the sample slowly to RT, a mixture of perovskite (*Pm-3m*) and scheelite (*I4_1_/a*) phases is obtained, as observed in the NPD pattern displayed in Figure 2. This annealing process of the reduced perovskite phase at 400 °C promotes a partial oxidation to the scheelite phase. This temperature was chosen because the transformation of the perovskite into a scheelite phase occurs in the temperature range between 370 and 450 °C, as described in a termogravimetric analysis reported in Ref. [10]. The thermal treatment in air at 400 °C for 1 h was, then, carefully chosen to lead to a mixture of the two, oxidized and reduced phases, and therefore to demonstrate the absence of intermediate phases between perovskite and scheelite oxides. Therefore, Figure 2 shows the Rietveld plot of this mixture of SrMo_0.9_Mg_0.1_O_4-δ_ scheelite and SrMo_0.9_Mg_0.1_O_3-δ_ perovskite, coexisting after heating in air the SrMo_0.9_Mg_0.1_O_3-δ_ perovskite sample at 400 °C for 1 h and cooling down to RT. The three series of reflection markers in Figure 2 correspond to the SrMo_0.9_Mg_0.1_O_4-δ_ scheelite (first series), SrMo_0.9_Mg_0.1_O_3-δ_ perovskite (second series) and V from the sample holder (third series). The structural parameters for both SrMo_0.9_Mg_0.1_O_3-δ_ perovskite and SrMo_0.9_Mg_0.1_O_4-δ_ scheelite phases are included in Table 1, together with the agreement factors of the profile refinement.

The coexistence of both oxidized and reduced phases, with no intermediate oxides or decomposition stages, demonstrates that the transformation between perovskite and sheelite takes place directly in a topotactical way. The scheelite structure was refined in the *I4_1_/a* space group, with Z = 4. Sr atoms are located at 4*b* (0, 1/4, 5/8), Mo and Mg are located at random at 4*a* (0, 1/4, 1/8) and oxygen atoms O1 at 16*f* (x, y, z) sites, whereas the SrMo_0.9_Mg_0.1_O_3-δ_ perovskite can be Rietveld-refined in the cubic *Pm-3m* space group at room temperature, Z = 1. In this space group, Sr atoms are located at 1*b* (½, ½, ½) position, Mo and Mg atoms are distributed at random at 1*a* (0, 0, 0) sites and the O oxygen atoms are placed at 3*d* (½, 0, 0) position. A small oxygen deficiency was observed at room temperature after refining the occupancy factors of the oxygen atoms.

**Figure 3:**
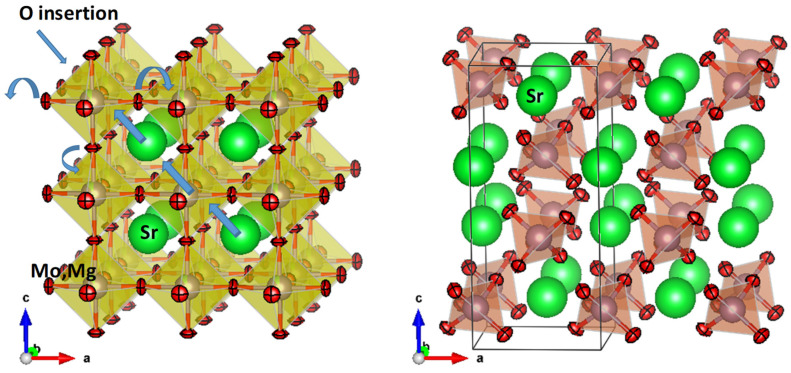
Views of the perovskite (**left**) and scheelite (**right**) crystal structures approximately along the (010) crystallographic plane, suggesting the topotactical transformation upon oxidation and shifts of metal and oxygen atoms.

Figure 3 shows a view of the reduced perovskite and the oxidized scheelite crystal structures. A topotactical transformation (in this case oxidation) implies that a basic structural framework remains almost unchanged during the transition. Figure 3 highlights the topological relationship existing between both structural types. For the SrMo_0.9_Mg_0.1_O_3-δ_ perovskite arystotype, the Sr cations are surrounded by 12 oxygens in dodecahedral coordination, whereas the Mo/Mg cations exhibit an octahedral coordination, with six closer oxygen atoms, which are shared with neighboring octahedra along the three directions of the crystal. It is noteworthy that the (110) plane shows a rectangular checker board-like arrangement where Sr and (Mo, Mg) alternate in the two directions.

Interestingly, the scheelite structure on the (001) crystallographic plane also exhibits a checker board-like distribution of metal cations. We observe that the perovskite can be simply transformed into the scheelite arrangement by shifting double rows of metal atoms, which is possible once the connectivity within the octahedral framework is broken. This mechanism can work at moderate temperatures via point defects. On the other hand, the octahedral oxygen coordination MoO_6/2_ sharing corners (corresponding to 3 oxygen atoms per Mo ion) can be converted into the tetrahedral environment, in isolated MoO_4_ units, present in the scheelite structure, with the topotactical insertion of one more oxygen per Mo ion (the oxidation process happening in air atmosphere), combined with shifts of the oxygen atoms.

It is interesting to calculate the volumetric effect of this transformation. For the scheelite phase the unit cell volume is 350.07 (2) Å^3^, for Z = 4, while for the perovskite, the uno-cell volume is 61.315 (4) Å^3^ for Z = 1. Normalizing to Z = 1, the perovskite structure is, by far, much more compact than the scheelite framework (87.51 Å^3^ per formula unit). This is related to the fact that, in the perovskite (well known as one of the most dense structures for metal oxides), the (Mo, Mg)O_6_ octahedra are sharing corners in a compact 3D framework, where the empty space is occupied by Sr ions. In the scheelite structure the (Mo, Mg)O_4_ tetrahedra are isolated from each other, with no common oxygens. For this reason, this is a much open structure that allows oxygen to diffuse easily across the crystal upon reduction; the (Mo, Mg) coordination polyhedra are thus connected to each other giving rise to the much more compact perovskite oxide.

Our observation from NPD data confirms that the transformation proceeds with no reaction intermediates or partial decomposition of the complex oxides with segregation of simple species. It only involves the required insertion of extra oxygen atoms from the atmosphere to complete the coordination of Mo ions, once the connectivity of the adjacent octahedra is broken in the perovskite framework, leading to isolated (Mo, Mg)O_4_ units in the scheelite structure. This mechanism, happening at moderate temperatures, is essential for the reversibility of the reduced-oxidized phases upon warming up the fuel cells, and warrants its cyclability in SOFCs, thus avoiding cracking problems or delamination of the anode from the electrolyte, as demonstrated in TEC investigations in Ref. [10].

## 4. Conclusions

The SrMo_1-x_Mg_x_O_3-δ_ perovskite, which perform as anode for SOFCs, is transformed by topotactical oxidation into a SrMo_1-x_Mg_x_O_4-δ_ scheelite. Both phases coexist at 400 °C in the presence of air, which was observed from neutron diffraction data. The crystal structures of perovskite and scheelite phases were successfully refined in the cubic *Pm-3m* and tetragonal *I4_1_/a* space groups. There were no reaction intermediates or partial decomposition, but the transformation proceeds directly by oxygen insertion and shifts of double rows of metal atoms. This happens at moderate temperatures as low as 400 °C, where the mobility of cationic species is very limited, demonstrating a tight topological relationship between both structural types.

## Figures and Tables

**Table 1:** Structural parameters of the coexisting reduced perovskite SrMo_0.9_Mg_0.1_O_3-δ_, defined in the cubic *Pm-3m* (No. 221) space group, Z = 1, and oxidized scheelite SrMo_0.9_Mg_0.1_O_4-δ_ oxide, defined in the tetragonal *I4_1_/a* (No. 88) space group, Z = 4, after the Rietveld refinement from NPD data collected at RT (λ = 1.594 Å).

RT	SrMo_0.9_Mg_0.1_O_3-δ_	SrMo_0.9_Mg_0.1_O_4-δ_
a (Å)	3.9433 (2)	5.3930 (2)
c (Å)	-	12.0364 (7)
V (Å^3^)	61.32 (1)	350.06 (3)
Sr 1*b* (½,½,½)/4*b* (0, 1/4, 5/8) ^§^		
B_iso_ (Å^2^)	0.87 (1)	0.69 (9)
f_occ_	1.00	1.00
Mo, Mg 1*a* (0, 0, 0)/4*a* (0, 1/4, 1/8)^§^		
B_iso_ (Å^2^)	1.5 (1)	0.011 (1)
Mo/Mg f_occ_	0.894 (1)/0.108(1)	0.894 (1)/0.108 (1)
O1 3*d* (½, 0, 0)/16*f* (x, y, z) ^§^		
x	-	0.2407 (6)
y	-	0.1158 (4)
z	-	0.0414 (3)
β_11_ *	-	114 (10)
β_22_ *	-	−0.43 (7)
β_33_ *	-	17 (2)
β_12_ *	-	88 (8)
β_13_ *	-	10 (3)
β_23_ *	-	−0.16 (4)
B_iso_/B_eq_ (Å^2^)	2.55 (9)	0.61 (1)
f_occ_	0.97 (2)	0.95 (2)
Reliability factors **	-	-
χ^2^	7.11	7.11
R_p_ (%)	4.08	4.08
R_wp_ (%)	5.61	5.61
R_exp_ (%)	2.12	2.12
R_Bragg_ (%)	6.34	4.38

^§^ The Wickoff sites correspond to the perovskite and scheelite phases, respectively. * Anisotropic Betas (×10^4^). ** The X^2^, R_p_ and R_wp_ are identical since they refer to the profile. Only the Bragg R factors are individualized for each phase.

## Data Availability

The original contributions presented in this study are included in the article. Further inquiries can be directed to the corresponding authors.
